# Effects of Recreational GHB Use and Multiple GHB-Induced Comas on Brain Structure and Impulsivity

**DOI:** 10.3389/fpsyt.2020.00166

**Published:** 2020-04-02

**Authors:** Filipa Raposo Pereira, Minni T. B. McMaster, Arnt Schellekens, Nikki Polderman, Yvon D. A. T. de Vries, Wim van den Brink, Guido A. van Wingen

**Affiliations:** ^1^Department of Psychiatry, Amsterdam Neuroscience, University of Amsterdam, Amsterdam UMC, Amsterdam, Netherlands; ^2^Amsterdam Brain and Cognition, University of Amsterdam, Amsterdam, Netherlands; ^3^Department of Psychiatry, Radboud University Medical Centre (Radboudumc), Nijmegen, Netherlands; ^4^Nijmegen Institute for Scientist Practitioners in Addiction (NISPA), Nijmegen, Netherlands,

**Keywords:** gamma-hydroxybutyric acid, gamma-hydroxybutyric acid-induced comas, neuroimaging, substance use disorders, diffusion imaging, rape drug, impulsivity, corpus callosum

## Abstract

**Background and Aims:**

The regular use of gamma-hydroxybutyrate acid (GHB) can induce GHB-induced comas. Other substance use disorders are associated with alterations in brain structure and impulsivity. Here we aim to investigate if these are also modulated by either regular GHB use or GHB-induced comas.

**Methods:**

In a sample of human males, structural and diffusion neuroimaging data were collected for 27 GHB users with ≥4 GHB-induced comas (GHB-Coma), 27 GHB users without GHB-induced comas (GHB-NoComa), and 27 polydrug users who never used GHB (No-GHB). The structural brain parameters were analyzed macroscopically using voxel-based morphometry and microscopically using tract-based spatial statistics (TBSS) and tractography. Impulsivity was assessed with the Barrat Impulsivity Scale.

**Results:**

In comparison to the other two groups, the GHB-Coma group showed a higher fractional anisotropy in the body of the corpus callosum and a lower mean diffusivity in the forceps minor (*i.e.*, whole-brain TBSS analysis). No macrostructural differences nor microstructural differences, as assessed with tractography, were observed. The GHB-Coma group also reported higher impulsivity, which was more strongly associated with white matter volume and fractional anisotropy in tracts involved in impulse control (post-hoc analysis). GHB use per se was associated neither with differences in brain structure nor with impulsivity.

**Conclusions:**

The results suggest that multiple GHB-induced comas, but not GHB use per se, are associated with microstructural alterations in white matter and with higher self-reported impulsivity, which in turn was associated with white matter tracts involved in impulse control.

## Introduction

Since it was first synthesized in the 1960s, gamma-hydroxybutyrate acid (GHB) has been regularly used for different therapeutic purposes (1–4). Over the last three decades, however, the unique profile of GHB, combining stimulant and sedative effects, has contributed to its appeal as a recreational drug (1, 3, 4). The appealing effects of the drug start with euphoria, relaxation, and sexual arousal, readily evolving into a state of sedation and altered consciousness when higher doses are used (1–6). This intangible stimulant–sedative shift is dangerously associated with poor control of dosage and effect duration, which creates a high risk for overdosing (including GHB-induced coma), and can lead to tolerance and addiction (2–5). Despite the low prevalence of GHB use (last year’s prevalence was 0.1–13% worldwide), the number of GHB users seeking treatment for drug withdrawal and GHB addiction is rising, and GHB overdose ranks as the fourth most common drug related overdose in European emergency rooms (2, 4, 6–8).

GHB-induced comas are among the most common manifestations of GHB overdose, with many chronic heavy users experiencing on average more than 10 lifetime episodes (1–5). These are transient but deep comas that often reach Glasgow Coma Scale scores as low as 3 (totally unresponsive) (1, 2, 4, 5). Regardless, the absence of “hangover” after recovery of consciousness contributes to the erroneous idea among recreational users that GHB use is safe and harmless (1, 2, 4, 5). However, we recently showed that heavy GHB use with multiple GHB-induced comas is associated with differences in cognition and affect, which were linked to the abnormal activation of the prefrontal cortex (PFC) and the limbic regions (*e*.*g*., the hippocampus), and their functional connectivity with the temporal–parietal lobe regions involved in perception and attention (9–11). To a smaller extent, GHB use itself was also associated with altered resting state functional connectivity between the executive and the default mode networks (12).

Despite the above evidence, nothing is known about the effects of recreational GHB use and multiple GHB-induced comas on brain structure. On succinic semialdehyde dehydrogenase (SSADH) deficiency, a condition known to induce an abnormal accumulation of GABA and GHB in the brain, gray matter atrophy and white matter myelin alterations in the PFC, insula, limbic system (*e*.*g*., hippocampus), and the parietal-occipital cortex have been observed (13, 14). These are brain regions rich in GHB-binding sites and particularly sensitive to GHB-induced neurotoxicity, as shown studies on rodents (15–18). Furthermore, substance use disorders and in particular alcohol-use dependence (another GABAergic dependence), have been associated with substantial alterations in gray matter regions that are often linked to impulse control (*i*.*e*., orbital-frontal cortex, anterior cingulate cortex, medial frontal gyrus, and insula) (19–23). Moreover, alterations in white matter tracts implicated in reward processing and inhibitory control have been observed in both substance use disorders and conditions involving altered states of consciousness (*i*.*e*., inferior frontal-occipital fasciculus, IFOF; inferior longitudinal fasciculus, ILF; uncinate fasciculus, UF; cortical spinal tract, CST; internal capsule, corona radiate, superior longitudinal fasciculus, SLF; cingulum; or corpus callosum, CC) (19, 20, 22–29). Such alterations might be the structural correlate underlying the high levels of impulsivity that are often comorbid with these conditions (2, 19, 20, 22–25, 30).

This study aimed to investigate the effects of recreational GHB use and multiple GHB-induced comas on impulse control and brain structure. Self-reported impulsivity was measured with the Barratt Impulsivity Scale (BIS). The macrostructural differences were assessed with voxel-based morphometry analysis (VBM) of structural magnetic resonance imaging (sMRI) data, and diffusion-weighted imaging (DWI) data were investigated with a univariate whole-brain level tract-based spatial statistical analysis (TBSS) or with a region-of-interest (ROI) probabilistic tractography analysis (TrackVis), focused on the ILF, IFOF, and UF, for the assessment of microstructural differences. To distinguish between the effects of recreational GHB use as such and multiple GHB-induced comas, three different groups of participants were recruited: (1) GHB users who had ≥4 GHB-induced comas, (2) GHB users who never had a GHB-induced coma, and (3) polydrug users who never used GHB. We tested the following hypotheses:

- Regular recreational users of GHB who had multiple GHB-induced comas show higher impulsivity and macrostructural and microstructural brain alterations when compared to GHB users who never had a GHB-induced coma and to polydrug users who never used GHB.
- Regular recreational users of GHB who never had a GHB-induced coma show higher impulsivity and macrostructural and microstructural brain alterations when compared to polydrug users who never used GHB.

## Materials and Methods

### Participants

The participants (*n* = 81) were recruited for this cross-sectional study through addiction centers in The Netherlands, flyers, internet advertisements, and snowball sampling. Three different groups of male participants, matched on age and education level, were included: 27 GHB users with ≥4 GHB-induced comas (GHB-Coma), 27 GHB users without GHB-induced comas (GHB-NoComa), and 27 polydrug users who never used GHB (No-GHB). The criteria considered in this study were a result of self-reported parameters and urine tests. The inclusion criterion for the GHB-Coma group was >4 GHB-induced comas (to increase the contrast with GHB-NoComa group). The inclusion criterion for the GHB groups was the use of GHB ≥25 times within the 2 years preceding this assessment. The overall inclusion criteria were age (between 18 and 40 years), native Dutch speaker, and male gender (since the majority of GHB users are males) (4, 5, 9). The polydrug use criteria consisted in the co-use of alcohol, nicotine, cannabis, cocaine, any other stimulants (amphetamines, khat, and methylphenidate), ecstasy, ketamine, and/or sedatives (benzodiazepines). Abstinence from recreational drugs for at least 24 h preceding the initiation of this study was required from all of the participants. The overall exclusion criteria were a history of epilepsy, general anesthesia within the 2 years preceding the study, a contra-indication for functional magnetic resonance imaging (fMRI) scanning (*e*.*g*., metal objects in the body or head injury), any coma unrelated to GHB use, and currently under treatment for narcolepsy with cataplexy (since the treatment may involve the use of medication based on GHB). The participants excluded due to low-quality scans were two GHB-Coma and two no GHB cases, respectively. The study procedures were explained prior to the assessments and written consent was obtained from the participants. This study was performed in accordance with the Helsinki Declaration principles (7^th^ revision, 2013) and the Medical Research Involving Human Subjects Act (WMO, 1998) and approved by the Medical Ethics Review Committee of the Academic Medical Centre (ethical protocol number: METC 2014_172) (31).

### Procedure

The data herein presented were part of a study assessing the effects of chronic GHB use and GHB-induced comas in the human brain. It entailed a urine test, self-reporting questionnaires (*i.e.*, substance use habits, negative affect, and impulsivity), and structural and functional imaging scans collected as follows: sMRI, resting state (fMRI), episodic memory (fMRI), DWI, working-memory (fMRI), and emotion identification (fMRI). Outside the scanner, the participants performed digitized neuropsychological tests concerning verbal memory, spatial memory, intra-/extra-dimensional set shifting, and probabilistic reversal learning. In this report, we will only present data on impulsivity and brain structure. Other findings have been presented elsewhere (9–12).

### Questionnaires and Cognitive Testing

To assess the use of recreational drugs other than GHB, the participants completed the MATE 2:1 substance use questionnaire (32). The Dutch version of the adult reading test was used to assess premorbid intellectual functioning, considered a proxy for intelligence quotient (IQ) (33). Individual differences in impulsivity were assessed with the self-reported BIS (34, 35). This scale consists of six first-order factors (attention, cognitive instability, motor, perseverance, self-control, and cognitive complexity), each comprising three to seven items presented in random order. Each item is scored from 1 to 4, from never to always feeling a certain way, respectively (35). The scores of all items are then summed up per factor, where higher scores indicate higher impulsivity levels and lower scores indicate lower impulsivity levels (35).

### Statistical Analysis of Demographic and Clinical Data

Demographic and clinical data were analyzed with the SPSS24 software (IBM Software Analytics, New York, USA). Normally distributed data were evaluated through ANOVA. If not normally distributed, the data were transformed in order to obtain a normal distribution or were evaluated with non-parametric tests (**Tables 1** and **2**). The GHB use groups were tested for differences in daily dose (ml/day), days of using GHB in the preceding month, months of daily use, and total exposure as defined by years of use × daily dose. All of the groups were tested for differences in co-use of alcohol, nicotine, cannabis, cocaine, stimulants (amphetamines, khat, methylphenidate), ecstasy, ketamine, and sedatives (benzodiazepines). This was performed by assessing the self-reported measures of drug use considered in the MATE 2:1 questionnaire and represented here by a computed variable of total exposure to each substance (*i*.*e*., years of weekly use × daily dose; **Table 2**). The study groups were assessed for differences in impulsivity (BIS subscale; **Table 1**) (35).

**Table 1:** Demographic and behavioral data.

	GHB-Coma (*N* = 27)		GHB-NoComa (*N* = 27)		No-GHB (*N* = 27)		Difference
	Mean	± SD	Mean	± SD	Mean	± SD	P*
**Age**	25.60	5.43	26.22	4.58	27.76	9.31	0.506^a^
**Educational level**	6.56	1.61	6.81	1.18	6.64	1.41	0.799^a^
**Premorbid IQ**	90.20	10.30	97.63	7.52	93.88	8.32	0.027c,b^1,^*
**Daily dose of GHB (ml/day)**	48.16	41.09	17.87	11.17	–	–	< 0.001^b,^*
**Days of GHB use last 30 days**	12.96	13.23	2.85	2.16	–	–	0.039^b,^*
**Months of daily GHB use**	24.64	43.69	0.12	0.37	–	–	0.001^b^
**BIS attention**	12.72	2.46	10.96	2.55	11.92	2.55	0.049
**BIS cognitive instability**	7.68	2.01	6.59	2.00	6.04	1.74	0.013
**BIS motor impulsivity**	16.48	3.44	15.07	3.64	15.07	3.64	0.129
**BIS perseverance**	7.56	1.69	7.85	1.46	8.16	1.40	0.376
**BIS self-control**	14.80	2.58	12.59	3.17	12.56	1.94	0.005^b,c,2,3,^*
**BIS cognitive complexity**	12.84	2.90	11.48	2.28	11.81	2.55	0.103

SD, standard deviation; a, ANOVA; b, Mann–Whitney U; c, Kruskal–Wallis. ^1^Post-hoc Mann–Whitney U: premorbid IQ_GHB-Coma < GHB-NoComa, p = 0.008. ^2^Post-hoc Mann–Whitney U: self-control_GHB-Coma > GHB-NoComa, p = 0.012. ^3^Post-hoc Mann–Whitney U: self-control_GHB-Coma > No-GHB, p = 0.002.

**Table 2:** Exposure to recreational drugs (MATE2.1).

Exposure to recreational drugs							
	GHB-Coma (*N* = 27)		GHB-NoComa (*N* = 27)		No-GHB (*N* = 27)		Difference
	Mean	SD	Mean	SD	Mean	SD	*p*^a^
**Alcohol**	4.98	11.89	11.94	23.76	12.57	35.55	0.258
**Nicotine**	105.35	137.90	40.31	61.70	42.13	86.48	0.082
**Cannabis**	5.09	9.00	3.36	5.45	3.68	6.13	0.876
**Cocaine**	1.84	5.24	0.20	0.50	0.03	0.12	0.045^2,3,*^
**Stimulants**	3.63	7.84	0.57	2.15	0.16	0.39	0.003^2,3,*^
**Ecstasy**	2.10	5.00	0.09	0.32	0.41	1.38	0.013^1,*^
**Ketamine**	0.17	0.47	0.22	0.87	0.06	0.20	0.519
**Sedatives**	1.65	7.78	0.16	0.80	0.00	0.00	0.001^1,2,*^

SD, standard deviation. a, Kruskal–Wallis. ^1^Post-hoc analysis Mann–Whitney U: GHB-coma > GHB-NoComa; ecstasy, p = 0.005; sedatives, p = 0.010. ^2^Post-hoc analysis Mann–Whitney U: GHB-Coma > No-GHB; cocaine, p = 0.014; stimulants, p = 0.002; sedatives, p = 0.001. ^3^Post-hoc analysis Mann–Whitney U: GHB-NoComa > No-GHB; cocaine, p = 0.050; stimulants, p = 0.007. *p < 0.05.

### Image Acquisition

Diffusion-weighted and structural images were collected with a 3.0-T Ingenia scanner with a 32-channel head coil (Phillips Medical System, Best, The Netherlands). T1-weighted structural images (sagittal acquisition; voxel size: 1.0 × 1.0 × 1.0 mm^3^; flip angle: 9°; field of view: 256 × 240 mm^2^) were acquired with a magnetization-prepared rapid gradient echo sequence for spatial normalization purposes. Diffusion-weighted images were acquired in 32 isotropic directions in order to test white matter abnormalities. Each image consisted of 48 transverse slices (TE: 92 ms; voxel size: 2.5 × 2.5 × 2.5 mm^3^; flip angle: 90°; matrix: 94 × 94; b-value= 1,000 s/mm; 30 diffusion-weighted directions).

### Structural MRI Preprocessing and Analysis

Preprocessing of structural data was conducted using the Computational Anatomy Toolbox 12 (CAT12; v.1363, http://dbm.neuro.uni-jena.de/cat12/) as implemented on Statistical Parametric Mapping software (SPM12 v.7219, Welcome Trust Centre for Neuroimaging, http://www.fil.ion.ucl.ac.uk/spm/software/spm12). Prior to preprocessing, the origin was manually set to the anterior commissure. T1-weighted images were segmented into gray matter, white matter, and cerebral spinal fluid after bias-field correction to remove non-uniformities in intensity, normalized into Montreal Neurologic Institute (MNI152) space, and smoothed using a Gaussian kernel of 8 mm at full-width half-maximum. In addition, total intracranial volume (TIV) was estimated to correct for differences in total brain size.

A voxel-based analysis of gray and white matter images thresholded at a tissue probability of 0.15 was performed to assess the macrostructural volume differences between groups. Since the differences between the groups were found in IQ and exposure to cocaine, stimulants, ecstasy, and sedatives, these were introduced as nuisance covariates in a general linear model (IQ as linear variable; co-exposure to the four substances as dummy variables). The number of variables representing co-exposure to other drugs in which group differences were observed was adapted to the sample size considered for each neuroimaging method of analysis. TIV was used to correct for differences in brain volume across subjects (36).

### DTI Preprocessing

Preprocessing of DTI data was conducted with the FMRIB Software Library 5.0.10 (FSL; Analysis Group, FMRIB, Oxford, UK; www.fmrib.ox.ac.uk/fsl) (37). Pre-processing consisted in eddy-current correction of potential distortions induced by gradient coils and head motion artefacts, individual non-brain tissue removal with the brain extraction tool, estimation of the diffusion tensor model at each voxel with the DTIFit tool, generating fractional anisotropy (FA; degree of diffusion directionality), mean diffusivity (MD; average diffusivity rate), axial diffusivity (AD; diffusion rate along the main axis of the tensor), and radial diffusivity (RD; diffusion rate transverse to the main axis of the tensor) scalars (23–25, 38). The diffusion tensor was then assessed for voxel-wise microstructural differences in white matter, at whole-brain with TBSS, or at three *a priori* defined ROI (tracts) with streamline tractography analysis.

### Whole-Brain TBSS Analysis

Whole-brain white matter differences were assessed with TBSS (FSL) as follows: (1) non-linear alignment of individual pre-processed FA images to a common FMRIB58 FA brain template (MNI152, 1 × 1 × 1 mm space), (2) averaging of aligned images into a mean FA map of all individual FA images, (3) creation of a mean FA skeleton map and a mean FA mask by computing a white matter tract skeleton into the mean FA map, and (4) creation of an all-skeletonized FA map (preserving only the central voxels of tracts common to all subjects) thresholding the mean FA skeleton map at 0.2 (39). All FA skeletonized data were submitted to voxel-wise statistical analyses. IQ, exposure to cocaine, stimulants, ecstasy, and sedatives were introduced as nuisance covariates as described previously. The same analysis was repeated for MD, AD, and RD scalars by projecting the aligned individual images of each participant into the created mean FA skeleton map.

### Tractography ROI Analysis

Tractography was performed on the ILF, the IFOF, and the UF based on their involvement in impulse control and proximity to regions where functional alterations were associated with recreational GHB use (9–11, 19, 23, 24, 26). Integrity differences in these tracts were assessed with streamline tractography using the TrackVis software (v0.6.01;Wang R, Wedeen VJ, Athinoula A. Martinos Center for Biomedical Imaging, Massachusetts General Hospital, 2015, www.trackvis.org/download/). For compatibility reasons, data were transformed from nifti to dtk format, and the tensor orientation was flipped around the z-axis (diffusion toolkit; v0.6.4.1; Wang R, Wedeen VJ, Athinoula A. Martinos Center for Biomedical Imaging, Massachusetts General Hospital, 2016). 3D virtual dissections were performed per participant, per tract and per hemisphere, by manually delineating two ROIs per tract on the FA maps of each participant based on established references of regions crossed by specific tract bundles (40, 41). An exclusion ROI was hand-drawn when needed for undesirable streamline elimination. The following ROIs were drawn on white matter: (a) ILF_ROI1: sagittal plane (first coronal slice, posterior edge of the cingulum), entire occipital area from the parietal–occipital sulcus medially to the temporal–occipital junction laterally; (b) ILF_ROI2: sagittal plane (most posterior slice, separation between the temporal and frontal lobes), entire ipsilateral–temporal lobe; (c) IFOF_ROI1: sagittal plane (first coronal slice between the posterior edge of the cingulum and of the parietal–occipital sulcus), entire occipital region posterior to the parietal–occipital sulcus and the temporal–occipital junction; (d) IFOF_ROI2: sagittal plane (first coronal slice, leveled at the anterior edge of the CC genu), entire external capsule; and (e) UF_ROI1: identical to IFOF_ROI2. UF_ROI2: sagittal plane (most posterior coronal slice, separation between the temporal and frontal lobes), entire anterior temporal lobe extending prior to the tract’s u-shape section. When all tracts were identified, the mean FA, MD, AD, and RD data were extracted and assessed with Kruskal–Wallis non-parametric tests; family-wise-error (FWE) rate was corrected (*p*_FWE_ < 0.05) for multiple comparisons using a height threshold of *p* < 0.001 and a Bonferroni correction to account for the multiple ROIs tested.

### Statistical Analysis of Voxel-Wise Neuroimaging Data

For VBM and TBSS analyses, multiple voxel-wise comparisons were corrected with threshold-free cluster enhancement permutation tests (TFCE; *p*_FWE_ < 0.05) (36). Two orthogonal planned contrasts were used to test our hypothesis considering GHB-induced coma (contrast a; GHB-Coma group vs. GHB-No Coma group and No-GHB group) or GHB use per se (contrast b; GHB-NoComa group vs. No-GHB group).

## Results

### Demographic and Clinical Characteristics

There were no significant group differences in age (mean ± SD = 26.51 ± 6.90) and education level (mean ± SD = 6.67 ± 1.40; **Table 1**) in any of the different sample sizes that had to be considered for each neuroimaging method of analysis (due to the quality of data acquisition). However, premorbid IQ was significantly lower in the GHB-Coma than in the GHB-NoComa group (*p* = 0.008). On average, the GHB-Coma group used GHB in higher daily doses (*U* = 139, *p* < 0.001), more frequently in the preceding month (*U* = 229, *p* = 0.018), and daily during more months (*U* = 192, *p* = 0.001) when compared to the GHB-NoComa group. The co-use of recreational drugs was also significantly different between groups (*p*_FWE_ < 0.05; **Table 2**). Overall the GHB-Coma group used more ecstasy and sedatives than the GHB-NoComa group and more cocaine, other stimulants, and sedatives than the No-GHB group, while the GHB-NoComa group used more cocaine and other stimulants than the No-GHB group. Such differences were considered in our neuroimaging analysis by introducing them as nuisance covariates. Finally, the GHB-Coma group showed lower attention and self-control and higher cognitive instability than the other two groups. However, when the analysis was Bonferroni corrected for multiple comparisons (*α* =.05/6 = 0.008), only self-control [higher impulsivity; *X*^2^(2) = 10.437, *p* = 0.005] remained statistically lower in the GHB-Coma group compared to the GHB-NoComa group and the No-GHB group (*p* = 0.012 and *p* = 0.002, respectively).

### Neuroimaging Data

**Figure 1:**
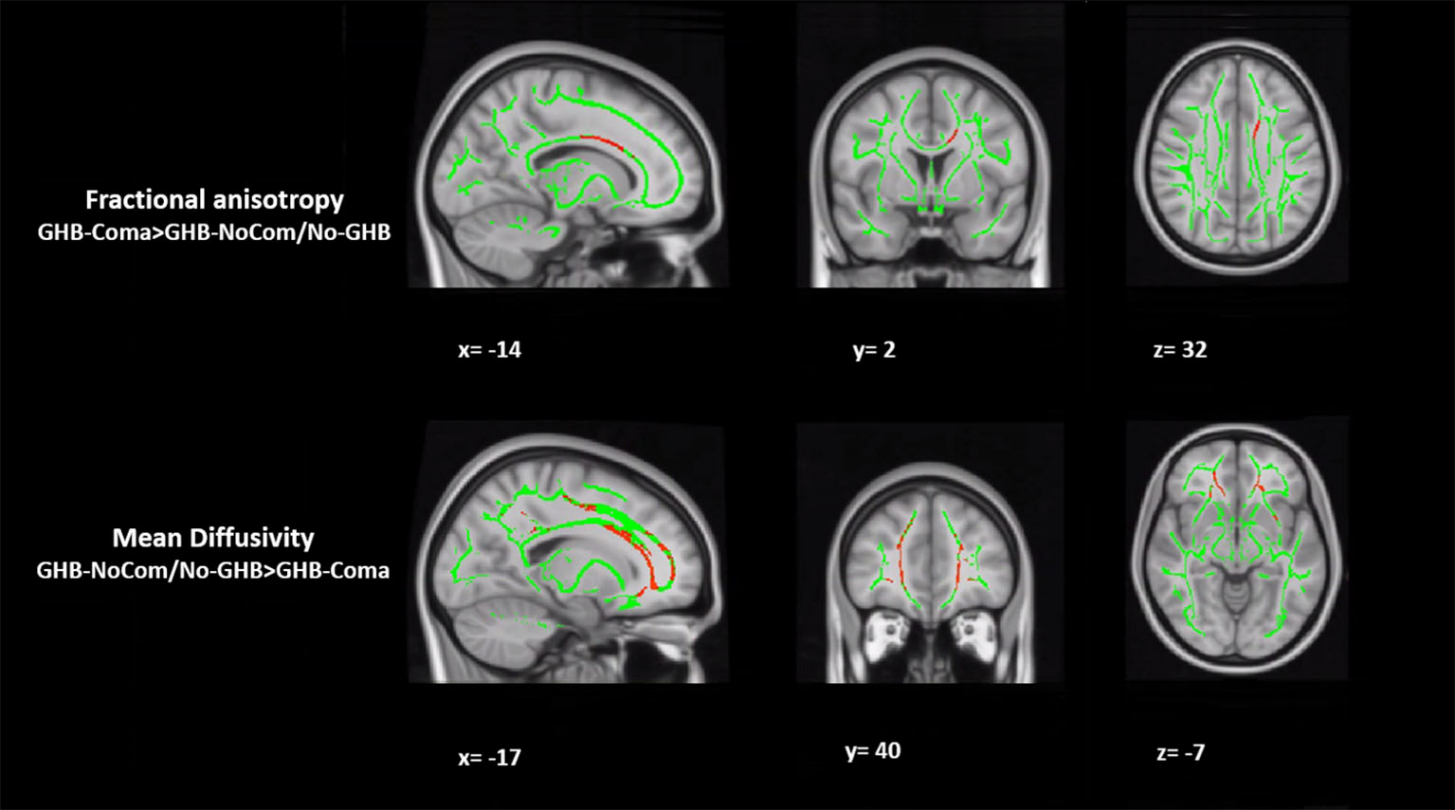
Tract-based spatial statistical (TBSS) analysis of white matter. The figure represents the sagittal, coronal, and axial brain planes of white matter skeleton (in green), with representations of increased fractional anisotropy of the body of the corpus callosum and decreased mean diffusivity of the forceps minor in the GHB-coma group, when compared with the GHB-no coma group and the No-GHB group (in red). [family-wise-error (FWE; p < 0.05) corrected using the threshold-free cluster enhancement (TFCE)].

When testing for the effect of GHB-induced coma, in comparison with the other two groups, the GHB-Coma group showed higher FA in the body of the CC and lower MD in the forceps minor (tracts identified with DTI-JHU atlas; **Table 3**; **Figure 1**) (40). No differences in the brain macrostructure nor in tractography were found. When testing for the effect of GHB use per se, no significant differences were observed. During the analysis of the neuroimaging data, the following participants were excluded per neuroimaging method due to excessive head movement inside the scanner or insufficient brain coverage: GHB-Coma group: three VBM, two TBSS, and two tractography; GHB-NoComa group: one VBM, two TBSS, and four tractography; No-GHB group: two VBM, four TBSS, and four tractography. These differences in the sample number were considered throughout the analysis of demographic and clinical data and values were adjusted whenever necessary. However, in **Table 1** and **Table 2**, the data presented concerns the totality of the study sample.

**Table 3:** White matter tracts where increased fractional anisotropy and decreased mean diffusivity were observed in the GHB-Coma group when compared with the GHB-NoComa group and the No-GHB group.

Microstructural differences in white matter integrity associated with multiple GHB-induced comas						
Regions	L/R	MNI coordinates				
		X	Y	Z	Voxels	*P*
**Increased FA coma > others**						
Body of corpus callosum	L	-14	2	32	369	0.042
**Decreased MD coma > others**						
Forceps minor	L	-17	40	-7	8,188	0.03

R, right; L, left; FA, fractional anisotropy; MD, mean diffusivity; MNI: Montreal Neurological Institute. Differences resulting from a tract-based spatial statistical (TBSS) analysis of white matter; family-wise-error (FWE; p < 0.05) corrected using threshold-free cluster enhancement (TFCE).

### Correlations

**Figure 2:**
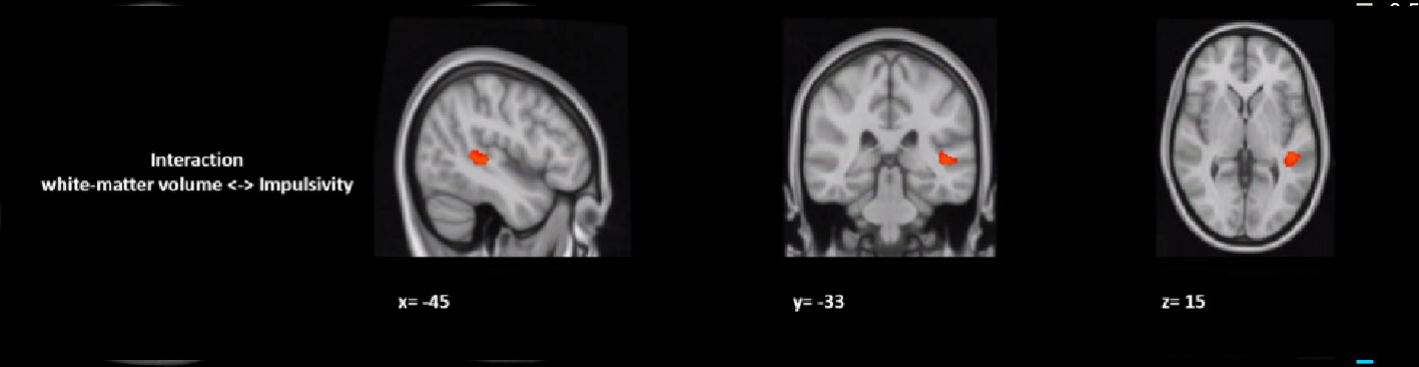
Group by impulsivity interaction analysis between white matter volume (macrostructure) and self-control. The figure represents the sagittal, coronal, and axial brain representations of a white matter region in the superior longitudinal fasciculus, shown to strongly interact with the self-control levels of the GHB-coma group when compared to the GHB-no coma group and the no GHB groups. [family-wise-error (FWE; *p* < 0.05) corrected using the threshold-free cluster enhancement (TFCE)].

**Figure 3:**
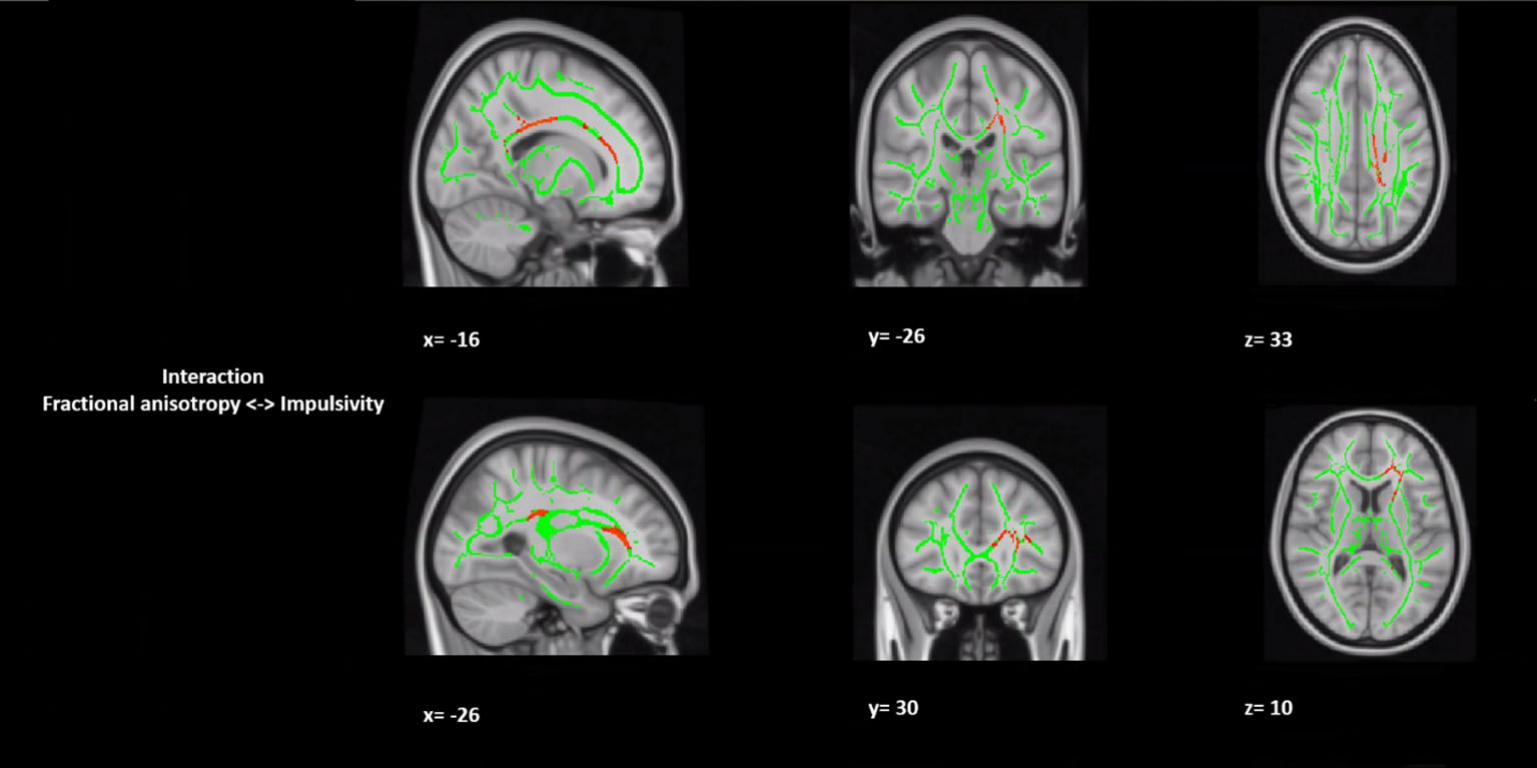
Group by impulsivity interaction analysis between white matter integrity (microstructure) and selfcontrol. The figure represents sagittal, coronal, and axial brain representations of the body of the corpus callosum and the uncinate fasciculus, shown to strongly interact with the self-control levels of the GHB-coma group when compared to the GHB-no coma group and the no GHB group. In green, representation of the white matter skeleton; in red, regions where interaction was different between groups. [family-wise-error (FWE; *p* < 0.05) corrected using the threshold-free cluster enhancement (TFCE)].

In a *post hoc* analysis, we assessed whether group differences in impulsivity were associated with macrostructure or microstructure (DTI indices from TBSS or tractography analyses) data by using a group-by-impulsivity interaction controlled for IQ, cocaine, other stimulants, ecstasy, sedatives, and TIV (19, 22, 23). Macrostructurally (VBM), when compared with the other two groups, the GHB-Coma group showed a stronger correlation with white matter volume of the posterior SLF (**Table 4** and **Figure 2**). When the four DTI indices assessed with TBSS were considered, the same interaction analysis showed a stronger correlation between impulsivity and FA of the left cingulum and the left UF in the GHB-Coma group when compared with the other two groups (**Table 4**; **Figure 3**). Lastly, a similar interaction was assessed with an ANOVA on the four DTI indices (per tract and per hemisphere) obtained from tractography assessment. This showed similar tendencies for the FA and AD of the left UF, which did not survive correction for multiple comparisons (**Table 4**). No interaction effect was observed between gray matter and impulsivity.

**Table 4:** List of the white matter tracts resulting from a group-by-impulsivity interaction analysis (according to different neuroimaging techniques, *i.e.*, VBM, TBSS, and tractography) showing significant interactions with impulse control in the GHB-oma group when compared with the GHB-NoComa group and the No-GHB group.

White matter regions interacting with self-control (impulsivity) found in association with the effect of multiple GHB-induced comas						
Regions	L/R	MNI coordinates				
		X	Y	Z	Voxels	*P*
**VBM** **White matter volume <-> impulsivity**						
Superior longitudinal fasciculus (III)	L	-45	-33	1.5	425	0.034^a^
**TBSS** **FA <-> impulsivity**						
Body of corpus callosum	L	-16	-26	33	1,455	0.033^a^
Uncinate fasciculus	L	-26	30	10	1,228	0.037^a^
**Tractography** **FA <-> impulsivity**						
Uncinate fasciculus	L	–	–	–	*F* = 3.528	0.065^b^
**Tractography** **AD <-> impulsivity**						
Uncinate fasciculus	L	–	–	–	*F* = 3.534	0.065^b^

VBM, voxel-based morphometry; TBSS, tract-based spatial statistics; R, right; L, left; FA, fractional anisotropy; AD, axial diffusivity; MNI, Montreal Neurological Institute. ^a^Analysis family-wise-error (FWE; p < 0.05) corrected using threshold-free cluster enhancement (TFCE). ^b^Analysis FWE (p < 0.05) uncorrected for multiple comparisons

## Discussion

GHB-induced comas seem to be associated with anatomical differences exclusively in the white matter at a microstructural level. Furthermore, the GHB-Coma group reported higher impulsivity than the other two groups, which strongly interacted with the FA of the left corpus callosum body and the left UF microstructurally and macrostructurally with the left SLF. No morphological brain differences were associated with GHB use per se, indicating that the structural brain abnormalities were primarily related to GHB-induced comas.

When compared with the other two groups, a voxel-wise TBSS analysis showed increased FA in the body of the CC and decreased MD in the forceps minor (part of the CC) of the GHB-Coma group. In contradiction of our first hypothesis, increased FA and decreased MD are general indicators of white matter integrity. This suggests that the anatomical alterations observed might have been present already before the occurrence of GHB-induced comas and represent a risk factor for the onset and development of heavy chronic use of GHB (22, 26, 42, 43). However, a similar directionality in FA and MD has been associated with different acute and subacute unconscious periods (hours to weeks) as a consequence of cytotoxic edema (cellular swelling linked to hypoxia) that results from factors such as myelin injuries (42–46). In alcohol use disorders (another GABAergic drug of abuse), the same directionality has also been suggested to be a result of myelin dysregulation, which was correlated with severity of alcohol drinking (47). Together these findings suggest that both or either the number of GHB-induced comas or the heavy doses taken chronically by the GHB-Coma group contribute to the anatomical alterations observed. Nevertheless, only AD or RD are sensitive biomarkers to the axonal or myelin nature of white matter alterations (respectively) and these parameters were not associated with GHB-induced comas (42–46). Thus, the myelin nature of the observed white matter abnormalities remains hypothetical. Furthermore, the CC and the forceps minor (branch of the CC) are tracts responsible for inter-hemispheric communication. Disruption in their integrity has been linked to deficits in affect dysregulation, associative memory, goal-directed behavior, or impulse control (48–50). Interestingly, parts of these fasciculi parallel functional connectivity pathways where alterations were previously associated with the GHB-Coma group while performing similar cognitive processes, suggesting that the alterations found might represent a structural correlate to such functional deficits (9–12).

In contrast to these TBSS findings, no alterations in white matter integrity were found with tractography. However, TBSS assesses local integrity, whereas tractography assesses mean integrity along the entire tract, suggesting that in the GHB-Coma group, microstructural differences in white matter only occur at a more local level (19, 26, 51, 52). Moreover, transient unconsciousness is mostly associated with subtle injuries often observed only in white matter, of which relatively crude methods such as structural MRI lack the sensitivity to detect (23, 25, 26, 53, 54). This was also the case in this study where no macrostructural differences in gray or white matter were observed between the groups. Thus, the occurrence of macroanatomical differences is likely related to a more severe exposure to GHB and/or to multiple GHB-induced comas.

Lastly, since impulsivity is a common comorbidity of substance use disorders (particularly of alcohol use dependence) and a lasting effect of transient unconsciousness, we decided to compare the level of impulse control between the groups (19, 21–26, 29, 53). In the mentioned conditions, gray and white matter alterations have been observed in regions linked to inhibitory control (19, 21–26, 29, 53). Here, although no alterations were observed in gray matter, we found a strong interaction in the GHB-Coma group between self-control and the SLF (macrostructurally) and with the FA of the CC and the UF (microstructurally), tracts that are highly implicated in impulse control. Furthermore, considering the structural alterations observed in the CC of the GHB-Coma group, it is reasonable to assume the involvement of this brain region in self-control. The interaction between the CC and lower self-control in the GHB-Coma group might thus be a neural correlate of the increased impulsivity in this group. Nevertheless, no data are available of the period prior to this study. Hence, this cross-sectional study cannot distinguish between impulsivity as a consequence of heavy GHB use or repeated GHB-induced comas and impulsivity as a risk factor for heavy GHB use. The same stands for the lower IQ observed in the GHB-Coma group when compared with the other two groups. Despite the fact that all of the participants were matched for education level (median/high level), the lower IQ observed in the GHB-Coma group suggests this to be a result of heavy use of GHB and/or the number of GHB-induced comas of this group. However, the cross-sectional nature of this study does not allow us to establish a causal link.

Moreover, it is important to consider the fact that the pre-frontal and limbic parts of the white matter tracts, where alterations were found in this study, are regions rich in GHB binding sites that have been shown to be highly sensitive to neurotoxicity induced by chronic GHB intake (as observed in animal studies) (19, 21–26, 29, 53). Moreover, GHB-induced comas have been compared to a state of pharmacological-induced unconsciousness and might also represent a source of neurotoxicity based on their capacity to induce hypoxia and consequent oxidative stress in such sensitive regions (4, 55, 56). The GHB-Coma group chronically used high concentrations of GHB and had multiple GHB-induced comas. Therefore, the observed outcomes might be partly explained by GHB-induced neurotoxicity resulting from either one or both of these factors, which in turn might potentiate the development of GHB use disorders. However, no structural scans or information on impulsivity was collected before the first GHB-induced coma had occurred and it cannot be excluded that anatomical and/or impulsivity differences were risk factors for the start of GHB use. Finally, the lack of structural group differences associated with GHB use per se might be related to the lack of a healthy control group and does not mean that differences with healthy (drug-naive) controls would not exist. Also, even the doses used by the GHB-NoComa group are still higher than the typical therapeutic doses used for narcolepsy and alcohol use disorders. Therefore, patients using medically prescribed GHB should not worry about neurotoxicity.

The multimodal assessment of brain structure was a particular strength of this study. It allowed the characterization of macrostructural and microstructural brain differences at the whole brain or at ROIs (23, 25, 26, 51, 52). The inclusion of two control groups is another strength that allowed us to distinguish between the effects of GHB use per se and GHB-induced comas. However, this study also has limitations. First, the exclusion of females does not allow the generalization of these results to female GHB users (4, 5). Second, premorbid IQ was lower in the GHB-Coma group when compared with the other two groups. This might have been an *a priori* trait that biased the motivational system of this group towards the immediate reward provided by GHB use. However, all groups were matched for education level and, therefore, the lower IQ in the GHB-Coma group is most likely a result from the repeated GHB-induced comas. Notwithstanding, IQ was used as a covariate in the analysis. Moreover, the last few years have witnessed an increase in the use of GBL (*i*.*e*., pro-drug of GHB). However, the participants included in this study reported solely the use of GHB in its absolute form (57). Another limitation is the fact that the assessment of GHB use was based solely on self-reported data. However, objective markers of GHB in blood or urine are nearly impossible to obtain as the metabolization and excretion is rapid and no standardized procedures and cut-off points are currently available for hair analysis (57, 58). There were also significant differences in the co-use of recreational substances between the groups, and despite their introduction as nuisance covariates to control for their influence, residual confounding cannot be excluded. Finally, due to its cross-sectional nature, this study addresses neither causality nor directionality.

In conclusion, multiple GHB-induced comas, but not GHB use per se, are associated with microstructural alterations in parts of the corpus callosum linked to goal-directed behavior, associative memory, and affect regulation. In the GHB-Coma group, these alterations might be suggested as the structural basis for the brain activity differences observed during the processing of such cognitive control functions. Moreover, the GHB-Coma group reported low self-control, which was found to interact with regions responsible for impulse regulation. Hence, considering the increasing treatment demand for GHB use disorders, defining the neurobiological substrates of comorbid impulsivity becomes of fundamental importance for the development of interventions that can normalize or improve the current treatments. Besides, the chronic use of high-doses of GHB with multiple GHB-induced comas represents a serious risk for brain impairment, and maximum treatment efforts are required, including relapse prevention using a combination of psychotherapy (*e*.*g*., MET/CBT) and pharmacotherapy (*e*.*g*., baclofen) (59, 60). Finally, it is of primordial relevance to integrate such findings in current awareness campaigns in order to change the common misleading perception among chronic users that GHB use is safe and that no harm is associated with GHB intoxication or GHB-induced comas.

## Data Availability Statement

All datasets generated for this study are included in the article.

## Ethics Statement

The studies involving human participants were reviewed and approved by the Medical Ethics Review Committee of the Academic Medical Centre. The patients/participants provided their written informed consent to participate in this study.

## Author Contributions

MM, WV, and GV designed the study. MM, NP, and YD collected the behavioral and neuroimaging data. AS contributed in the collection of the data. FR performed the neuroimaging and statistical analyses. FR and GV were responsible for the interpretation of the data. FR wrote the manuscript. FR, MM, WV, GV, YD, and AS have revised and approved the submission of the final manuscript.

## Funding

This research was funded and supported by the Ministry of Health, Welfare, and Sport of The Netherlands.

## Conflict of Interest

The authors declare that the research was conducted in the absence of any commercial or financial relationships that could be construed as a potential conflict of interest.

## Abbreviations

ANOVA, analysis of variance; AD, axial diffusivity; BIS, Barrat Impulsivity Scale; BET, brain extraction tool; CBT, cognitive behavioral therapy; CAT12, Computational Anatomy Toolbox 12; CC, corpus callosum; CST, cortical spinal tract; DWI, diffusion-weighted imaging; FWE, family-wise-error; FOV, field of view; FA, fractional anisotropy; FWHM, full-width half-maximum; fMRI, functional magnetic resonance imaging; GHB, gamma-hydroxybutyrate acid; IFOF, inferior frontal-occipital fasciculus; ILF, inferior longitudinal fasciculus; IQ, intelligence quotient; MPRAGE, magnetization-prepared rapid gradient echo; MD, mean diffusivity; MNI, Montreal Neurologic Institute; MET, motivation enhancement therapy; PFC, prefrontal cortex; RD, radial diffusivity; SPM, Statistical Parametric Mapping software; sMRI, structural magnetic resonance imaging; SSADH, succinic semialdehyde dehydrogenase; SLF, superior longitudinal fasciculus; TFCE, threshold-free cluster-enhancement permutation tests; TIV, total intracranial volume; TBSS, tract-based spatial statistics; UF, uncinate fasciculus; VBM, voxel-based morphometry.
